# Treatment of Basal Cell Carcinoma of the Lower Eyelid With High-Dose-Rate Brachytherapy

**DOI:** 10.7759/cureus.53067

**Published:** 2024-01-27

**Authors:** Catarina N Oliveira, Cláudia Viveiros, Catarina Travancinha, António Mota, Inês Pino, João Fonseca, Tiago Madaleno, Miguel Labareda, Filomena Santos, Susana Esteves

**Affiliations:** 1 Radiotherapy, Instituto Português de Oncologia de Lisboa Francisco Gentil, Lisbon, PRT; 2 Radiotherapy, Centro Hospilalar Barreiro Montijo, Barreiro, PRT; 3 Radiotherapy, Hospital CUF Descobertas, Lisbon, PRT; 4 Radiotherapy, Institut Jean-Godinot, Reims, FRA; 5 Medical Physics, Mercurius Health, Lisbon, PRT; 6 Radiotherapy, Hospital SAMS, Lisbon, PRT; 7 Clinical Investigation Unit, Instituto Português de Oncologia de Lisboa Francisco Gentil, Lisbon, PRT

**Keywords:** interstitial brachytherapy, high-dose-rate brachytherapy, eyelid, skin cancer, basal cell carcinoma

## Abstract

Objective

To report the outcomes with high-dose-rate (HDR) brachytherapy (BT) treatment in patients with lower eyelid basal cell carcinoma (BCC) and to evaluate the relationship between dosimetric parameters and acute and late toxicities.

Material and methods

A retrospective unicentric study with patients diagnosed with lower eyelid biopsy-proven BCC treated with HDR BT between January 2012 and December 2019. The prescribed dose was 36 Gy to 40 Gy in 9 to 10 fractions, twice daily, over five days. The primary endpoint was local control, and the secondary endpoints were acute and late toxicities, registered according to CTCAE v4.0. The cosmetic result was evaluated on a qualitative scale (the CAIB scale). Local control was calculated according to the Kaplan-Meier test. Two sample T-tests and a Wilcoxon signed-rank test were used to determine the association between dosimetric parameters and side effects.

Results

Fifty-eight patients with a median age of 76 years were included. Among these patients, 55.2% received adjuvant HDR BT and 44.8% received radical HDR BT. At a median follow-up of 44 months, there were four local relapses, achieving a probability of local control at four years of 95% and 100% in the adjuvant and radical groups, respectively. Acute toxicity occurred in 76% of patients with only one grade 3 event (radiation dermatitis). Late toxicity was present in 56%. Eight patients underwent treatment for grade 3 cataracts during follow-up. Cosmetic results were excellent or very good in 93% of patients. Acute conjunctival hyperemia is strongly associated with the dose received by the ocular globe (volumes of 0.1cc, 1cc, and 2 cc) (p<0.05).

Conclusion

Lower eyelid BCC treatment with interstitial HDR BT is associated with excellent local control, acceptable long-term side effects, and good cosmetic results.

## Introduction

Eyelid tumors represent 5%-10% of skin cancer, with basal cell carcinoma (BCC) accounting for 90% [1]. More than 50% of periocular BCC cases arise in the lower eyelid, often displaying local invasiveness. Management poses significant challenges, primarily attributed to the location and consequential impact on both functionality and cosmesis [2].

There are several treatment modalities available, and although Mohs micrographic surgery remains the gold standard, radiotherapy is an effective alternative [3]. Radiotherapy can be used in the adjuvant setting or as a first-line definitive treatment in selected cases where functional and cosmetic outcomes are a concern or in patients who are unsuitable for surgery. Indications for adjuvant radiotherapy are positive or close surgical margins, perineural invasion, and lymphovascular space involvement [4].

Brachytherapy (BT) can be delivered superficially through contact or using an interstitial application [5]. While low-dose-rate (LDR) BT is historically well established, the first case report with high-dose-rate (HDR) was only published in 2007 [6]. Initial concerns about toxicity and cosmetic outcomes associated with HDR BT were noted in the early years of its utilization, but subsequent published results have consistently demonstrated favorable results [7].

The present study aims to report the experience of an institution with HDR BT treatment in patients with lower eyelid BCC in terms of outcomes and to assess the association between dosimetric parameters and acute and late toxicities. This article was previously presented as a poster at the 10th European Congress on Head and Neck Oncology in March 2023.

## Materials and methods

Inclusion criteria and treatment characteristics

Unicentric retrospective study of patients with lower eyelid or inner canthus BCC treated with interstitial HDR BT with an ^192^Ir source between January 2012 and December 2019 in the Radiotherapy Department of Instituto Português de Oncologia de Lisboa Francisco Gentil, E.P.E. No patient had been previously treated with radiotherapy. All patients had a biopsy-proven BCC as well as written consent agreeing to the treatment with HDR BT. No patients were excluded, and data were collected by reviewing clinical records and available complementary exams.

**Figure 1:**
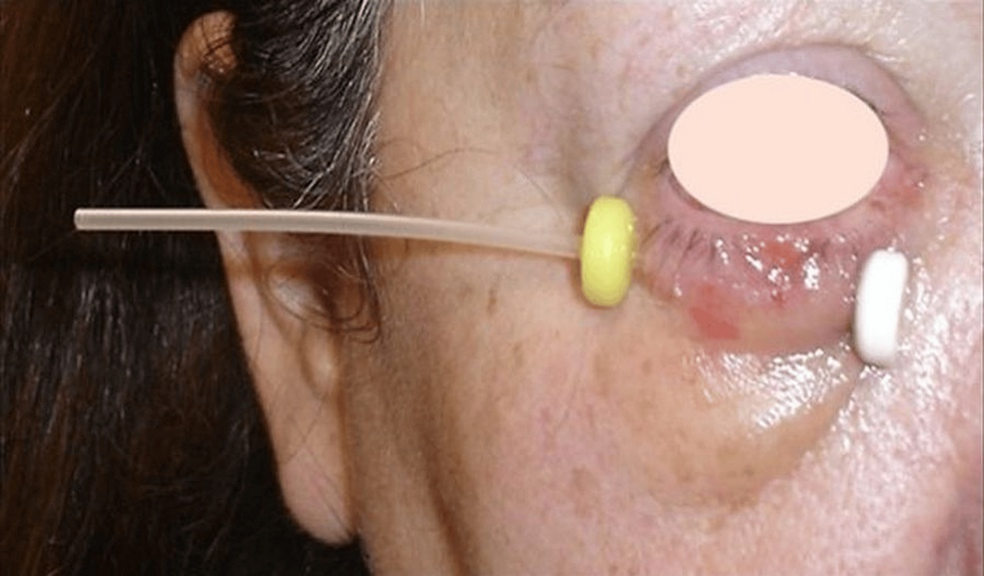
An example of an implant with a 6F Elekta catheter held still with one button in each extremity.

Regarding the application procedure, implantation was carried out according to the modified Paris system. The procedure was done under asepsis and local anesthesia. Eyelid skin cancer can be treated with a single-plane implant and generally with one single catheter at a depth of at least 5mm from the skin surface. If more than one catheter is needed, they are placed in a parallel position, spaced 10 to 15 mm from each other. After insertion, needles were replaced by 6F Elekta catheters held in proximity with plastic buttons (Figure 1).

**Figure 2:**
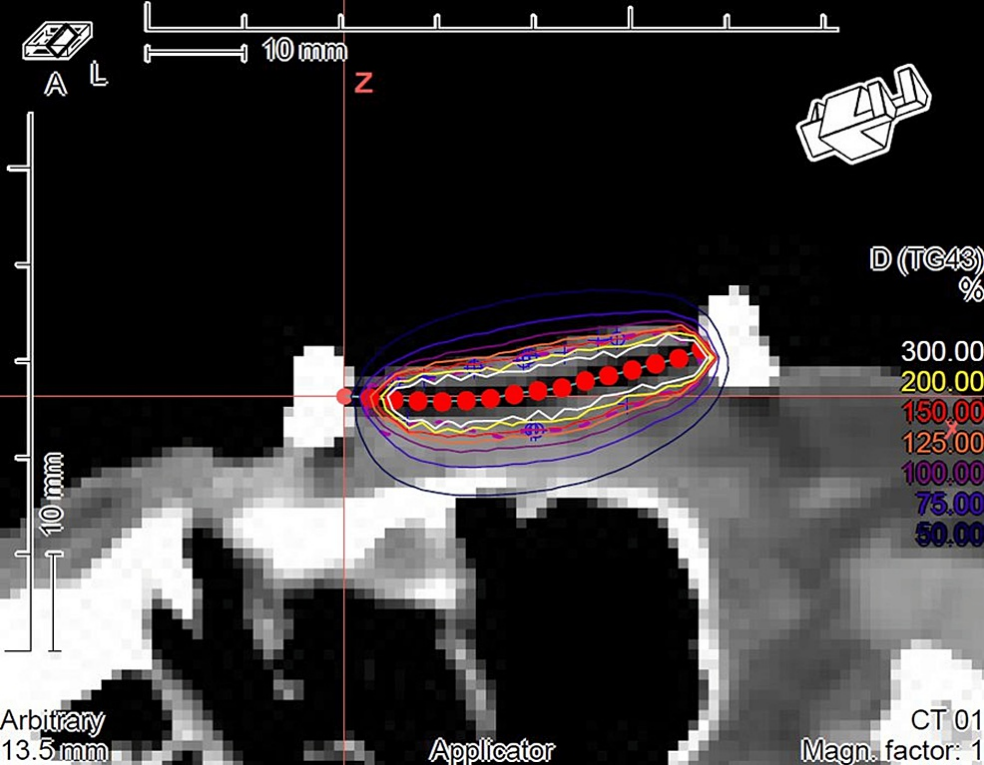
Example of dosimetry of interstitial high-dose-rate brachytherapy in basal cell carcinoma of the lower eyelid.

Computed tomography (CT) simulation scans were performed, acquiring 2mm slices, and exported to the treatment planning system (Elekta Oncentra® Brachy). Clinical target volume (CTV) and organs at risk (ipsilateral lens and eye) were contoured on CT axial slices. CTV was defined as gross tumor volume (GTV) plus a 0.5 to 1cm margin. Information on surgery, pathology, or other complementary exams was considered to better define the target. Dwell time dose optimization based on 3D CT images (Figure 2) was calculated.

The prescription dose was 40 Gy in 10 fractions (BED_10_ 56 Gy; BED_3_ 93 Gy) for macroscopic disease and 36 Gy in 9 fractions (BED_10 _50.4 Gy; BED_3_ 84 Gy) for microscopic disease. The dose volume parameter used to assess CTV coverage was D90. Treatment fractions were administered twice daily, at least 6 hours apart. Dose, volumes, and Total Reference Air Kerma (TRAK) were registered as indicated in ICRU (International Commission on Radiation Units and Measurements) 58 [8].

Treatment was delivered with Elekta MicroSelectron v2 HDR ^192^Ir Nucletron, and patients were instructed to look in the opposite direction of the eye that was being irradiated. Patients remained ambulatory between treatments. Analgesics and nonsteroidal anti-inflammatory drugs, as well as topical treatment with bacitracin and dexpanthenol, were given.

Statistical analysis and outcome assessment

Concerning the statistical analysis, the primary endpoint was local control, and secondary endpoints encompassed acute (up to six months after treatment) and late toxicities (more than six months after treatment), classified according to Common Terminology Criteria for Adverse Events version 4.0 (CTCAE v4.0). Cosmesis was evaluated on a qualitative scale (excellent, good, fair, and bad). No scale has been validated in this context; however, the Cosmesis Assessment following Interstitial Brachytherapy (CAIB Scale, Table 1) was used in our study, similarly to Laskar et al. [9].

**Table 1:** Cosmesis assessment criteria following interstitial brachytherapy (CAIB Scale). Score in cosmesis outcome: sum based on the presence or absence of late toxicity criteria displayed in the upper portion of the table. This table was adapted from Laskar SG et al. (2014) [9].

Criteria based on late toxicity	Present	Absent
Depigmentation of skin	score 1	score 0
Eyelid dysfunction	score 1	score 0
Dry eye	score 1	score 0
Keratitis	score 1	score 0
Cataract	score 1	score 0
Glaucoma	score 1	score 0
Cosmesis Outcome	Score (sum)	
Excellent	0	
Very good	1 - 2	
Fair	3 -4	
Poor	5 - 6	

Follow-up time was defined as the period of time since the end of treatment until the last obtainable revaluation consult or death. Local control was calculated according to the Kaplan-Meier test. Two sample T-tests and a Wilcoxon signed-rank test were used to determine the association between dosimetric parameters and the most frequent side effects. Results are also presented by frequency (number of patients and corresponding percentage), and a significance level was obtained for a p-value of 0.05.

## Results

A total of 58 patients were included. The median age was 76 years (39-89 years). The majority of tumors, accounting for 75.9% (n=44), were located in the lower eyelid, and the remaining 24.1% (n=14) in the inner canthus. Four patients (6.9%) had recurrent BCC. No tumor had invaded adjacent structures. BT was adjuvant to surgery in 55.2% of cases (n=32) and radical in the remaining patients. Tumor incomplete resection or positive margins were the main indications (n=24 or 75%) for adjuvant treatment (Table 2).

**Table 2:** Patients and tumor characteristics. Median age (range: minimum-maximum): years; n: number of patients (%: percentage). BT: Brachytherapy

Patients and tumor characteristics	n (%)
Age (years)	
Median (minimum to maximum)	76 (39-89)
Gender	
Male	30 (51.7%)
Female	28 (48.3%)
Tumor Location	
Lower eyelid	44 (75.9%)
Inner canthus	14 (24.1%)
Purpose of brachytherapy	
Radical BT	26 (44.8%)
Adjuvant BT	32 (55.2%)
Margin status	
Incomplete resection/Positive margins	24 (75%)
Not evaluable margins	3 (9.4%)

The median follow-up was 44 months (3.5 to 126.8 months), and at the time of data collection, there were four local relapses. The probability of four-year local control was 95% and 100% in the adjuvant and radical groups, respectively. The time to local relapse ranged between 34.1 and 93.9 months. There were seven unrelated deaths. According to the CAIB scale, cosmetic results were excellent in 51.7% (n=30) of patients and very good in 41.4% (n=24) (Table 3). Due to a lack of follow-up, the assessment of the cosmetic result was not possible for three patients (5.2%).

**Table 3:** Treatment outcomes. Median follow-up: months/years; n: number of patients (%: percentage).

Outcome	n (%)
Median follow-up in months/years (minimum to maximum)	44 (3.5-126.8)/3.7 (0.3-10.6)
Local recurrence	4 (6.9%)
Acute toxicity	44 (75.9%)
Late toxicity	31 (53.4%)
Cosmetic result	
Excellent	30 (51.7%)
Very good	24 (41.4%)
Fair	1 (1.7%)
Unknown	3 (5.2%)

Acute toxicity occurred in 44 out of 58 patients (75.9%). The most frequent acute adverse events were radiation dermatitis (n=26 or 44.8%, grades 1-2 and one grade 3 toxicity), conjunctival hyperemia (n= 18 or 31%, with only one grade 2 event), and eyelid edema (n= 14 or 24.1%). Less frequent acute adverse events include epiphora grade 1 (n=7 or 12.1%), pruritus grade 1 (n= 6 or 10.3 %), conjunctivitis grade 1 (n=4 or 6.9%) and local hematoma (n=3 or 5.2%) (Table 4).

**Table 4:** Acute toxicities. n: number of patients (%: percentage). CTCAE V4.0: Common terminology criteria for adverse events (CTCAE) version 4.0.

Acute toxicity (CTCAE V4.0 grade)	n (%)
Radiation dermatitis (grade 1-2)	26 (44.8%)
Conjunctival hyperemia (grade 1-2)	18 (31%)
Eyelid edema (grade 1)	14 (24.1%)
Epiphora (grade 1)	7 (12.1%)
Pruritus (grade 1)	6 (10.3%)
Conjunctivitis (grade 1)	4 (6.9 %)
Hematoma (grade 1)	3 (5.2%)
Hypopigmentation (grade 1)	2 (3.4%)
Radiation dermatitis (grade 3)	1 (1.7%)

Data concerning late toxicities was available for 55 patients. Overall, late toxicity occurred in 31 out of 55 patients (56.4%) (Table 5). The adjuvant BT subgroup had more late toxicity (n= 19 or 59.3%) than radical BT patients (n=12 or 46.2%). Epiphora grade 1 (n= 10 or 18.2%), conjunctival hyperemia grade 1 (n=10 or 18.2%), and hypopigmentation grade 1 (n= 9 or 16.4%) were the most frequent late toxicities.

**Table 5:** Late toxicities. n: number of patients (%: percentage). CTCAE V4.0: Common terminology criteria for adverse events (CTCAE) version 4.0.

Late toxicity (CTCAE V4.0 grade)	n (%)
Epiphora (grade 1)	10 (18.2%)
Conjunctival hyperemia (grade 1)	10 (18.2%)
Hypopigmentation (grade 1)	9 (16.4%)
Telangiectasia (grade 1)	3 (5.5%)
Fibrosis (grade 1)	3 (5.5%)
Eyelid edema (grade 1)	2 (3.6%)
Xerophthalmia (grade 1)	2 (3.6%)
Hypoesthesia	1 (1.8%)
Keratitis (grade 2)	1 (1.8%)

During follow-up, a total of eight patients (14.5%) developed grade 3 cataracts, five of which presented with bilateral cataracts and only three ipsilaterally to the treated site. None of these patients had a documented previous history of cataracts. The median dose at 0.1cc of the ipsilateral lens was 0.59Gy. An analysis of the correlation between dose to the lens and the development of ipsilateral cataracts was not feasible. This value was available in only 26 patients due to unretrievable dosimetric parameters from the planning system. Of those, three had grade 3 cataracts (doses of 16, 0.6, and 0Gy). The patient with D0.1cc to the lens of 16Gy developed ipsilateral cataracts.

**Figure 3:**
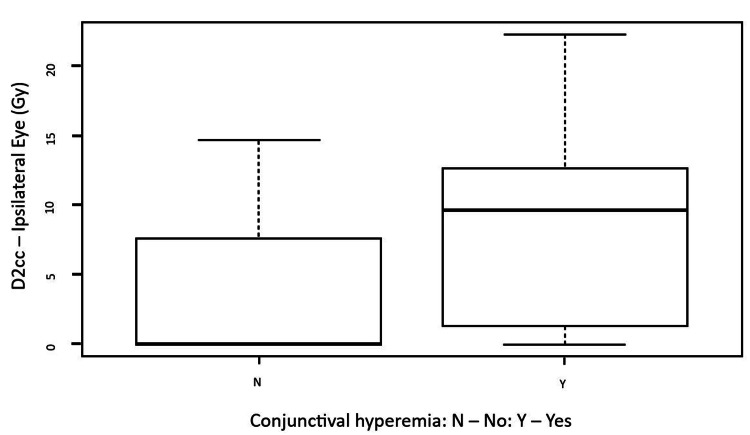
Vertical boxplot: minimal dose received in a 2cc volume of ipsilateral eye according to the presence of conjunctival hyperemia. D2cc: Minimal dose received in 2cc volume of ipsilateral eye (Gy: Gray); N: No; Y: Yes

There was no statistical association between CTV dosimetric parameters (V150, V200, and V300) and acute (p-values of 0.61, 0.83, and 0.75, respectively) and late toxicities (p-values of 0.34, 0.41, and 0.43, respectively). The most frequent acute toxicities, including radiation dermatitis (p-values of 0.34, 0.15, and 0.10), eyelid edema (p-values of 0.63, 0.62, and 0.45), and late toxicity hypopigmentation (p-values of 0.88, 0.90, and 0.56) were also tested. The dose received by the ocular globe (volumes of 0.1cc, 1cc and 2 cc) was strongly associated with the incidence of conjunctival hyperemia (p-value 0.01) (Figure 3).

## Discussion

Literature addressing interstitial HDR BT in the context of eyelid BCC is scarce. A total of nine results are indexed in MEDLINE, and the most relevant article considering sample size and follow-up time was published in 2014 [10]. This study included 52 patients with basal cell carcinoma and reported a local control of 96.7% in the overall cohort of 60 patients. Despite being favorable, the acute effects are overall worse in this study, with 80% acute conjunctivitis grade 2-3, which may be attributed to a higher prescription dose. Late effects are minimal (3% over grade 2), and the cosmetic outcome was unsatisfactory in only 8.3% of patients.

Our experience demonstrated a significant incidence of grade 3 cataracts, often bilateral, which could be due to a cohort with a median age of 76 years at diagnosis, corresponding to a baseline risk of age-related cataracts over 40% [11,12]. The available dosimetric data did not allow for correlation analysis in this study, but considering the low median dose at 0.1cc of the lens, the authors express reservations about affirming a clear association between the administered treatment and the development of cataracts. Nevertheless, the eye and lens are the main organs at risk in eyelid BT, and dosimetric parameters must be further evaluated in order to correlate with toxicities.

BT has a better cosmesis than EBRT (external beam radiation therapy), according to the Skin CanceR Brachytherapy vs. External Beam Radiation Therapy (SCRiBE) meta-analysis [13]. In our study, the cosmetic results were good to excellent in 93.1% of patients. However, there is no validated scale that allows comparison of cosmetic results in different studies, and the CAIB scale used in the present study does not consider some late toxicities like fibrosis, telangiectasias, or conjunctival hyperemia.

Other previous published studies [14,15] also demonstrate good local control with recurrence rates similar to Mohs surgery [16], although no prospective studies have compared the two approaches in the treatment of eyelid BCC. This study is subject to several limitations, including its single-center and retrospective nature and the lack of dosimetric parameters in some patients. Furthermore, extended follow-up would be ideal given the occurrence of relapse in one patient after 7.8 years and the reported median time to recurrence in the literature (3.9 years) [17]. Our findings are based on real-world data, and the results are likely applicable to broader populations. To summarize, our study supports that interstitial HDR BT (alone or adjuvant to surgery) for lower eyelid BCC is feasible and effective with good cosmetic results and low-grade toxicities. 

## Conclusions

Our study places interstitial HDR BT as a valuable treatment modality for lower eyelid BCC, with the potential to address anatomical and functional challenges as well as patient preferences. This technique is effective in the treatment of lower eyelid BCC, offering a valid alternative when the patient is unsuitable for surgery or cosmesis is a concern.

Although Mohs micrographic surgery remains the standard therapy, treatment with brachytherapy can also achieve excellent local control rates with acceptable acute and long-term effects, supported by the large proportion of patients with excellent or very good cosmetic outcomes.
